# Repeatedly Flashed Luminance Noise Can Make Objects Look Further Apart

**DOI:** 10.1177/2041669519855090

**Published:** 2019-06-20

**Authors:** Sabine Born

**Affiliations:** Faculté de Psychologie et des Sciences de l’Education, Université de Genève, Genève, Switzerland

**Keywords:** luminance noise, perceived separation, spatial vision, spatiotemporal factors

## Abstract

Luminance noise is widely used as mask in Experimental Psychology. But can luminance noise also affect where we perceive an object or change the perceived distance between objects? In this study, I investigated the effect of a repeatedly flashed luminance noise pattern on the perceived separation between two bars. Indeed, compared to conditions without dynamic luminance noise, the spacing between the bars was overestimated when the pattern flashed on-and-off in the background. The cause for this remarkably stable effect remains unknown. Potential relations to apparent motion, masking, attentional biases, and other visual illusions are discussed.

## Introduction

Luminance noise patterns are a widely used tool in Experimental Psychology. Often they are used as visual masks to reduce the visibility of a spatiotemporally overlapping target (see Breitmeyer & Öğmen, 2006). But can luminance noise also affect where we perceive an object or change the perceived distance between objects? Recently, we have found strong attraction of a briefly presented probe towards a stable reference when the probe was presented close in time to a flashed full-screen luminance noise pattern (Born, Krüger, Zimmermann, & Cavanagh, 2016; Zimmermann, Born, Fink, & Cavanagh, 2014). Further, a small jump of a peripheral target bar is harder to see when accompanied by flashed luminance noise (Zimmermann et al., 2014; Zimmermann, Morrone, & Burr, 2013). We suggested that masking of apparent motion may play a vital role in these effects: without motion providing a cue for separation, the spacing between two objects or the distance that a single object has covered with a jump may be underestimated.

Following up on this work, here I investigated the relationship between dynamic (i.e., repeatedly flashing) luminance noise and the perceived separation between two bars. If masking of apparent motion plays a role for the perceived separation between stimuli, then this may not only affect briefly flashed probes or the perception of a single target jump. It could also affect the perceived separation between objects in cyclic apparent motion displays, with objects jumping back and forth. On each trial, participants had to decide where the spacing between two bars was wider, in the pair presented to the left, or in the pair presented to the right. Surprisingly, when luminance noise flashed on-and-off in the background of one pair, the spacing between the two bars on that side was not under-, but overestimated (see supplementary material or https://osf.io/dc54x for a demo of Experiment 1b). Further, subsequent experiments showed that this effect was not due to masking of apparent motion, as it was also present for stimuli that did not jump back and forth.

## General Methods

### Participants

For each experiment, responses from 10 observers were considered for statistical analysis. If psychometric functions had poor fits (criterion: *R*^2^ < 0.7), the data set was excluded and an additional participant was run (one in Experiment 1b, four in Experiment 1d, one in Experiment 2a; see supplementary material or https://osf.io/2pxbw for individual data of all participants, including the six excluded data sets). Observers were first-year psychology students from the University of Geneva, participating for course credit, ranging between 17 and 51 years of age (mean: 23.0 years).

### Apparatus

Experiments were programmed in MATLAB (The MathWorks Inc., Natick, MA) using the Psychophysics and Eyelink toolboxes (Cornelissen, Peters, & Palmer, 2002; Kleiner, Brainard, & Pelli, 2007). Stimuli were displayed on ViewPixx or ViewPixx/3D screens (VPixx Technologies Inc., Saint-Bruno, QC, Canada). Fixation was monitored using an EyeLink1000 desk-mounted eye tracker (SR Research Ltd., Ottawa, ON, Canada). The participant’s head was stabilized by a chin rest at approximately 55 cm from the screen.

### Stimuli, Procedure, and Design

**Figure 1.**
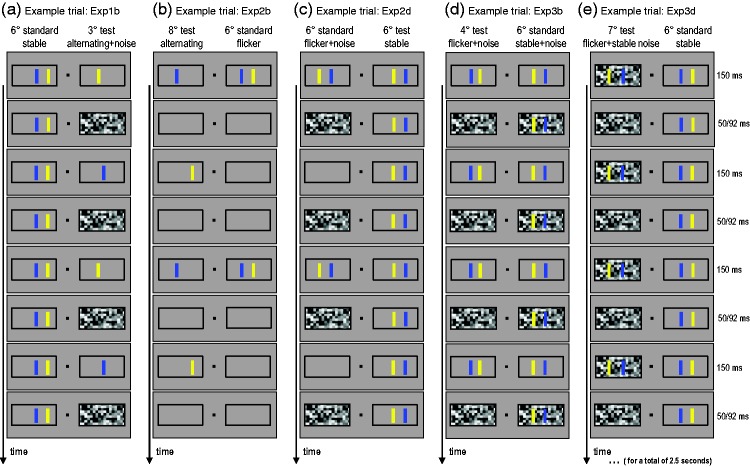
Examples of the stimulus stream in one trial for five different experiments. Participants fixated centrally and reported on which side the spacing between the blue and yellow bar appeared wider to them. In each experiment, two conditions were compared (e.g., Experiment 1b: stable bars vs. alternating bars with interleaved noise; see Panel (a)). On each trial, one of the conditions provided the standard with a fixed bar separation of 6°. On the other side, the spacing between the test bars was variable. Across trials, either condition could be assigned standard or test. A random horizontal position jitter was added to each pair. Flicker frequency in flicker + noise conditions differed, with bars only presented in between every second noise display in Experimental Series 2 (see Panels (b) and (c); to be comparable to the alternating conditions); or after every noise display in Experimental Series 3 (see Panels (d) and (e); to be more similar to the stable condition).

The timing of events in a trial is illustrated in Figure 1. Initially, the mask displays were presented for 50 ms (Series 1 and 2), later on they were displayed for 92 ms (Experiment 1d, Experiment 2d, Series 3).^1^ Stimuli were displayed on a light gray background (*x* = 0.27, *y* = 0.34, 51 cd/m^2^). On each trial, two pairs of bars were presented, one to the left and one to the right from central fixation. The bars (6° × 1.2° large) were presented inside outline boxes centered at 12° of eccentricity left and right from fixation (box size: 18° × 12°). Each pair consisted of one blue (*x* = 0.10, *y* = 0.10, 4 cd/m^2^) and one yellow bar (*x* = 0.36, *y* = 0.52, 76 cd/m^2^), to verify that participants always saw two bars on each side (otherwise, they were asked to press the down key; this happened on < 1% of trials). Whether the inner or outer bar was blue was random across trials and for each pair. Participants judged in which pair the bars were further apart from each other (key press: left or right). On each trial, there was one standard pair (always 6° apart), shown randomly either left or right; on the other side, the test bars were presented with a separation of 3°, 4°, 5°, 6°, 7°, 8°, or 9°. The pairs were not centered within the boxes, but an additional horizontal jitter of ±2° was added to both bars simultaneously, but independently for each pair and randomly varying across trials.

Bars were presented continuously (stable), flickering on and off, or alternating, giving the impression of one object moving back and forth (apparent motion). Stimuli could be presented with or without luminance noise, filling one or both of the boxes. The noise pattern consisted of grayscale squares of random luminance (same average luminance as the background) with a side length of 0.5°, flashing on and off. In the flickering and alternating conditions, the noise pattern was presented during the off phase of the bars; in the stable conditions, the bars remained superimposed on the noise pattern. In Experiment 3c and Experiment 3d, conditions with nonflashing, stable noise patterns were also tested. For better illustration of the different conditions, demo movies can be found in supplementary material or on https://osf.io/9hjf7. On each trial, the stimuli were presented for at least 2 s (five full cycles of back and forth apparent motion in the alternating conditions; 2.5 s in experiments with 92 ms masks). Participants were asked to keep fixating on a small square at screen center and to give their response after the stimuli had disappeared. Fixation was monitored by eye tracker and a feedback message was presented during the response phase if participants’ horizontal gaze coordinate deviated more than 2° away from the central square during stimulus presentation. Those trials were excluded from analysis.

Each participant compared two conditions, completing four blocks on the same comparison in a single 45-min session. Randomized across trials was the distributions of the two conditions across the two boxes (one left and the other right), either condition could be assigned the standard or test pair, and the standard of each condition was coupled with each of the seven test distances of the other condition. The combinations of this 2 (distribution left/right) ×2 (standard vs. test) ×7 (test distances) design were repeated three times in a block, resulting in 84 trials. Collapsed across left and right presentation, the four blocks thus provided 24 data points per combination.

### Analyses

**Figure 2.**
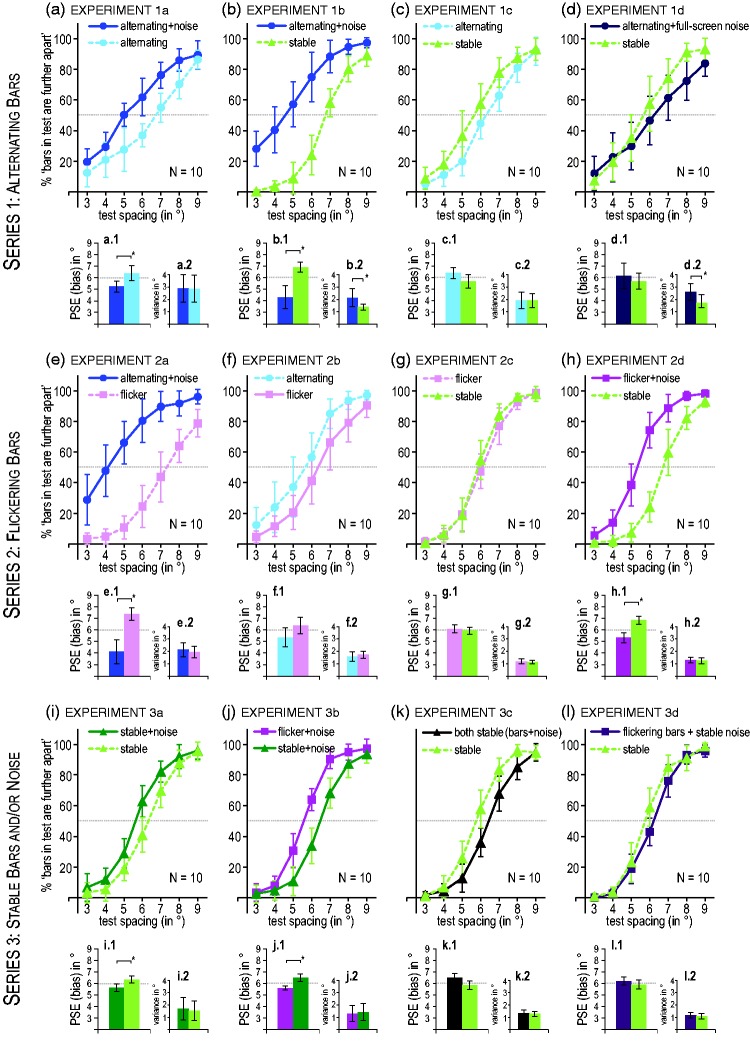
Results of all experiments. Line graphs: Percentage of trials for which the bars of the test pair of a given condition (color coded) were judged as further apart than the standard 6° pair of the other condition. Biases show when the two lines are markedly shifted. Bar graphs: average points of subjective equality as a measure of bias (left, X.1) and average variance as a measure of discriminability (right, X.2), derived from fits of cumulative Gaussian functions to individual data sets. Overestimations show in PSEs < 6°. All error bars: 95% confidence intervals of the means across participants.

Psychometric functions to each participant’s data were fit with cumulative Gaussians using a maximum-likelihood method in MATLAB. Descriptive statistics as well as 95% confidence intervals in Figure 2 were computed using IBM SPSS Statistics (IBM Corporation, Armonk, NY). Wilcoxon signed-rank tests as well as Bayes factors were calculated in JASP (JASP Team, 2018). Given the exploratory nature of the experiments, alpha levels are not corrected for multiple comparisons and two-tailed tests were run. For the Wilcoxon test statistic *W*, the smaller of the two values is reported; effect size is given by the matched rank biserial correlation *r*. Regarding the complementary Bayesian paired *t* tests, the JASP default values were used: Comparing the null hypothesis that effect size is 0 to an alternative hypothesis with effect size modeled by a Cauchy prior distribution centered on 0 and with a scaling factor of *r* = 0.71 (see Rouder, Speckman, Sun, Morey, & Iverson, 2009). The Bayes factor favoring either H0 (*BF*_01_) or H1 (*BF*_10_) is reported, depending on which of the two was larger than 1. All data, a description of the different analysis steps, as well as the JASP output can be found on https://osf.io/6fc2y.

## Experimental Series 1: Alternating Bars

### Results and Discussion

In the first experiment (Experiment 1a), observers judged the distance between alternating bars, comparing a condition with a luminance noise pattern presented during the off-phase of the stimuli to a condition without luminance noise. Based on our previous findings (Born et al., 2016; Zimmermann et al., 2014), my initial hypothesis was that masking of apparent motion results in underestimations of the spacing between bars in the condition with noise. Surprisingly, however, observers judged the spacing between bars as wider with noise (Figure 2(a)). The points of subjective equality (PSEs) for the test pairs (always compared to a standard pair of the other condition with a fixed distance of 6°) differed significantly, Wilcoxon signed-rank test: *W* = 6, *p* = .027, *r* = .78, *BF*_10_ = 2.18 (Figure 2(a.1)). Overestimations in the alternating + noise condition were much stronger, though, when compared to a baseline with completely stable bars and no noise (Experiment 1b), *W* = 3, *p* = .010, *r* = .89, *BF*_10_ = 18.56 (Figure 2(b)). In contrast, when comparing alternating bars without noise to the stable condition (Experiment 1c), apparent motion seemed to shrink the perceived distance between bars slightly (Figure 2(c)). However, the statistics were not conclusive: *W* = 13, *p* = .160, *r* = .53, *BF*_01_ = 1.22. Finally, I examined whether results depended on the noise patterns being confined to the boxes: Alternating bars embedded in a full-screen noise pattern (as we had used in our previous studies) were compared to stable bars, presented in a small window not filled by the pattern (Experiment 1d; not illustrated in Figure 1, but see demo on https://osf.io/r47wc). There were no overestimations of the alternating stimuli (Figure 2(d)). If anything, there were small underestimations for the larger bar spacings (see also Supplementary Series 101). The statistics did not point to a reliable difference, though, *W* = 23, *p* = .695, *r* = .16, *BF*_01_ = 2.78.

## Experimental Series 2: Flickering Bars

### Results and Discussion

The next comparisons tested whether the luminance noise influenced perceived spacing by reducing the perception of apparent motion. If so, then the alternating + noise condition should be comparable to a condition with flickering bars without noise (i.e., comparable stimulus energy, and likewise no apparent motion; Experiment 2a). Figure 2(e) shows, however, that the spacing between bars in the alternating + noise condition was still strongly overestimated when compared to flickering bars, *W* = 1, *p* = .004, *r* = .96, *BF*_10_ = 49.16. When comparing alternating bars without luminance noise to flickering bars (Experiment 2b), this strong bias was greatly reduced (see Figure 2(f) and statistics were not conclusive whether a difference persisted, *W* = 14, *p* = .193, *r* = .49, *BF*_01_ = 1.25. Flickering bars without noise did not produce any strong biases when compared to stable bars (Experiment 2c), either, *W* = 23, *p* = .695, *r* = .16, *BF*_01_ = 2.74 (Figure 2(g)). Quite remarkably, however, presenting the on-and-off flashing noise pattern with the flickering bars produced again robust overestimations when compared to stable bars without noise (Experiment 2d), *W* = 2, *p* = .006, *r* = .93, *BF*_10_ = 30.06 (Figure 2(h)). Thus, the bars presented with the noise pattern do not have to alternate to be judged as further apart (see also Supplementary Series 102).

## Experimental Series 3: Stable Bars or Noise

### Results and Discussion

Finally, the bars may not even have to flicker to produce overestimations in perceived spacing, but the on-and-off flashing noise pattern could be sufficient: When stable bars were presented on both sides (Experiment 3a), the spacing on the side with an additional noise pattern in the background was slightly, but significantly overestimated (Figure 2(i)), *W* = 4, *p* = .014, *r* = .86, *BF*_10_ = 2.54. Flickering bars produced overestimations compared to stable bars when both were presented with on-and-off flashing noise patterns (Experiment 3b; Figure 2(j)), *W* = 0 (i.e., all 10 participants show a difference in the same direction), *p* = .002, *r* = 1.00, *BF*_10_ = 39.68. In contrast, presenting the bars with perfectly stable (nonflashing) noise patterns did not produce any overestimations: When both, the bars and the pattern, were stable (Experiment 3c, Figure 2(k)) the two-tailed Wilcoxon test resulted in an almost significant difference, *W* = 10, *p* = .084, *r* =.86, pointing, however, to underestimations on the noise side. The Bayes factor was neither in favor of H1 nor H0, though, *BF*_10_ = 1.11. Finally, when flickering bars were presented on top of a stable noise pattern (Experiment 3d, Figure 2(l)), no obvious differences were observed compared to a condition with stable bars and no noise, *W* = 17, *p* = .322, *r* =.38, *BF*_01_ = 2.11.

## General Discussion

Summing up across all comparisons, an on-and-off flashing luminance noise pattern enlarged the perceived separation between two bars when the pattern was presented within a confining box around the stimuli; this effect was found when the bars alternated (Experiment 1a, Experiment 1b, and Experiment 2a), flickered (Experiment 2d; see also Supplementary Series 102), or were presented perfectly stable (Experiment 3a). Overestimations were not found with static noise patterns. While biases were remarkably strong (up to 1°–2° of bias in PSEs), the cause of the current effects remains unclear. In the following sections, I discuss several potentially related phenomena and mechanisms.

### Masking

Masking is the reduction in visibility of a target caused by another visual stimulus (Breitmeyer & Öğmen, 2006). Masking theories often distinguish between modulations of the transient or sustained response of the target (Breitmeyer & Öğmen, 2006; Macknik & Livingstone, 1998). According to Breitmeyer and Öğmen (2006, Chapter 5), transient channels primarily signal the presence, location, and motion of an object. Depending on the temporal dynamics, the signals of target and mask may either inhibit each other or integrate. Such a transient-sustained dual-channel model may provide a starting point for explaining the current effects, given that overestimations were only observed with on-and-off flashing noise patterns.

Many masking theories (e.g., Enns & Di Lollo, 2000) also assume that the perception of an object depends on a synthesis of feedforward afferent signals with feedback or re-entrant signals from higher level areas. Interestingly, Breitmeyer and Öğmen (2006, Chapter 5) postulate that changes to the input result in a transient inhibition of feedback signals, leading to a dominance of feedforward signals in perceptual synthesis. Up the visual hierarchy, the size of (population) receptive fields increases (Dumoulin & Wandell, 2008). Larger population receptive fields correlate with underestimations of the size of peripheral objects (Moutsiana et al., 2016), and potentially also perceived spacing. If the influence of higher level feedback is attenuated by repeatedly flashed noise, the final percept may be less influenced by areas with large receptive fields, resulting in relative overestimations.

### Visual References

Visual landmarks have been shown to slightly repulse the remembered location of objects (Werner & Diedrichsen, 2002). Others have reported that target objects may switch positions with distractors when masked with structured patterns (Mewhort, Huntley, & Duff-Fraser, 1993). Or the perceived location of a target may be pushed outside the boundary of a virtual surface defined by a metacontrast mask (Sigman, Sackur, Del Cul, & Dehaene, 2008). These examples stress the power of other visual objects to bias the perceived location of masked stimuli. Previously, we have found strong attraction of a masked probe towards a stable reference (Born et al., 2016; Zimmermann et al., 2014); that is, the opposite of the current bias. However, the probe was only briefly flashed (<50 ms). Also, we had used full-screen noise patterns, which did not produce overestimations in the current experiments, either (Experiment 1d; Supplementary Series 101). Key factors for over- or underestimations could therefore either be the overall disruptiveness of the noise pattern (coverage of the visual field) or whether parts of the pattern (e.g., its boundary) may act as a reference.

### Filled-Object and Other Illusions

Although the noise patterns had the same average luminance as the background, the two bars were of high contrast (and with opposite polarity) to the background. It is unclear whether any of these specifics were crucial for the current effects. It has been shown, for instance, that high contrast between objects and their background results in stronger perception of apparent motion (Anstis, 2003). Future experiments may be needed to disentangle whether contrast and polarity played a role in the current overestimations of perceived spacing.

On another note, the luminance noise pattern in the current experiments introduced structure in between the two bars. Similarly, in the classic Oppel-Kundt illusion (e.g., Mikellidou & Thompson, 2014), a horizontal line segment that is filled by several regularly spaced vertical lines is seen as longer compared to an equally long segment without the vertical lines. While the illusion demonstrates that filled spaces look bigger, an explanation still remains elusive (Mikellidou & Thompson, 2014). Note also that the Oppel-Kundt illusion does not depend on a dynamic background, while the current effects only emerged with flashing noise patterns.

### Response Bias

It can also not be excluded that the current findings are partly or fully due to response biases. Indeed, observers may shift their psychometric function through response bias without changes to the slope of the function (Morgan, Dillenburger, Raphael, & Solomon, 2012). Although possible, note that response biases are thought to emerge in situations of uncertainty: When unsure, participants may chose the side with the noise pattern for their answer. Thus, if solely due to response bias, the large shifts in the psychometric function would nevertheless indicate that the dynamic luminance noise patterns caused substantial uncertainty over quite a wide range of separations, despite the fact that bars were clearly visible. Future experiments may tap into this issue, for instance, by asking participants to indicate the pair with the smaller instead of the larger separation between the two bars.

### Attentional Modulations

The current results are also reminiscent of object-based warping (Vickery & Chun, 2010), a bias to judge two dots shown within an object as further apart, compared to two dots presented beside an object. As one explanation, the authors suggested that attention may select the object and spread across its entire surface. Spatial attention shifts may make a moving dot pattern appear larger (Anton-Erxleben, Henrich, & Treue, 2007), potentially also expanding the perceived spacing between the dots. Also, shifting attention towards a peripheral cue repulses a subsequently presented bar, making it look further away from the cue (Suzuki & Cavanagh, 1997). On the one hand, the current flashing noise patterns almost certainly attracted attentional resources. On the other hand, stimulus streams were presented for more than 2 s, recruiting sustained attentional resources. It has been suggested that sustained attention (in contrast to attentional shifts) produces underestimations of the perceived distance between objects within its focus (Liverence & Scholl, 2011). Thus, the attention literature does not provide clear predictions for the current setup, but an attentional influence cannot be ruled out.

## Supplementary Material

Supplementary material

Supplementary material

Supplementary material

Supplementary material

Supplementary material

Supplementary material

Supplementary material

Supplementary material

Supplementary material

Supplementary material

Supplementary material

Supplementary material

Supplementary material

Supplementary material

Supplementary material

Supplementary material

Supplementary material

Supplementary material

Supplementary material

Supplementary material

## Note

1 Series 1 and 2 were partly run in parallel; Experiments 1d and 2d were the first to be run on a new setup: For reasons of lab organization, the ViewPixx (100 Hz) was replaced by a ViewPixx/3D (120 Hz) screen. Due to a programming issue, this resulted in mask displays lasting for 92 ms instead of 50 ms. The longer duration was maintained in Experimental Series 3.
