# Supplementary material for: Repeatedly Flashed Luminance Noise Can Make Objects Look Further Apart
Source: Iperception. 2019 Jun 20;10(3):2041669519855090. doi: 10.1177/2041669519855090 (PMC6591532; doi:10.1177/2041669519855090)
Supplement: Supplementary material [file Supplemental_Material1.pdf]

## SUPPLEMENTARY MATERIAL: INDIVIDUAL SUBJECT DATA

### EXPERIMENTAL SERIES 1 : ALTERNATING BARS

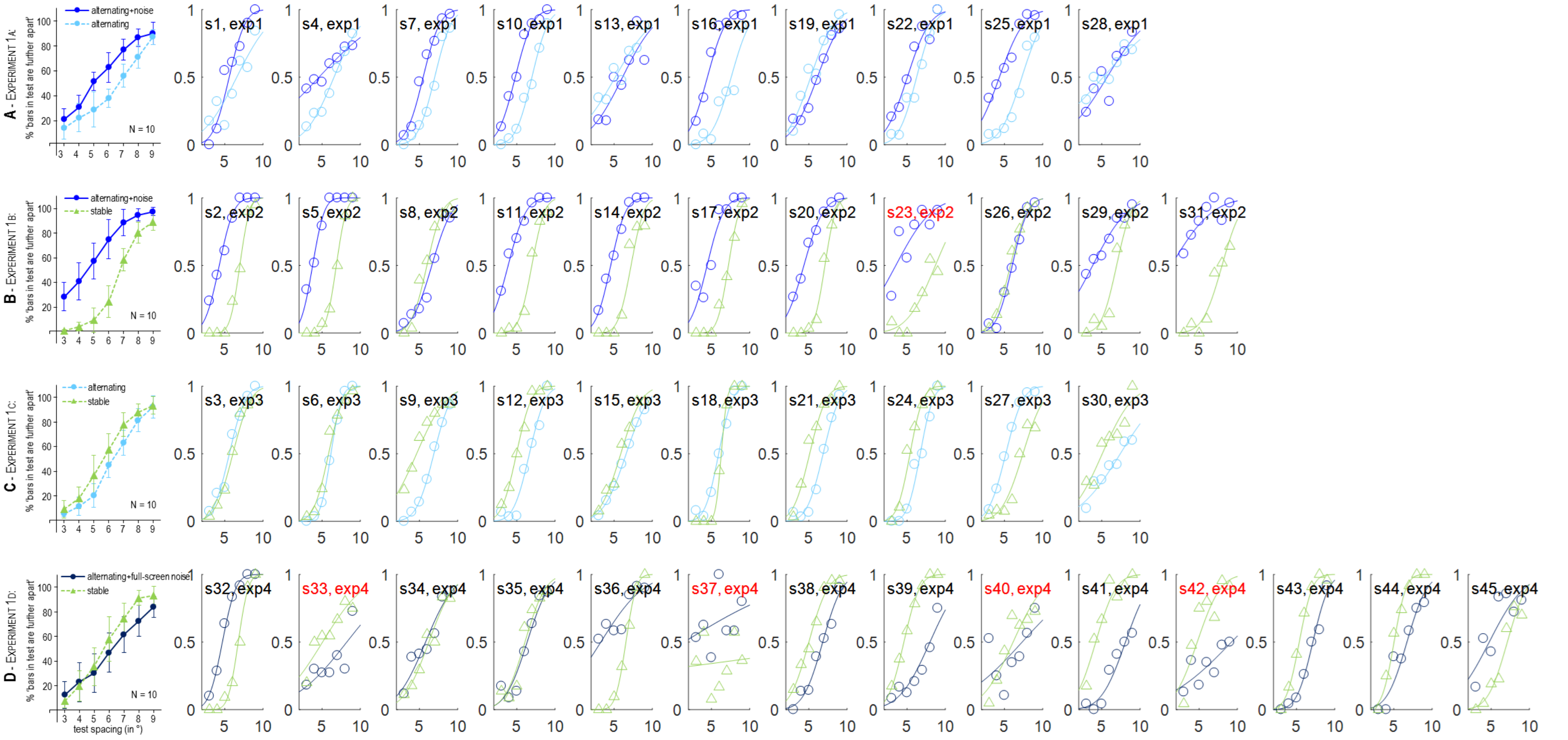


**Supplementary Figure S1.** Proportions of 'bars in test further apart' responses individually for all participants from Experimental Series 1, along with the psychometric functions fit to the data. Leftmost column: averaged data as reported in the manuscript. Participants whose data was excluded from analysis (due to $R^2$ of at least one of the fits < 0.7) are marked in red font. In Experiment 1b, this was the case for one participant (s23). Note, however, that this participant's pattern was similar to the averaged data. In Experiment 1d (last row), four participants were excluded. Just as for the remaining participants, data for the excluded participants was inconsistent.

## EXPERIMENTAL SERIES 2: FLICKERING BARS

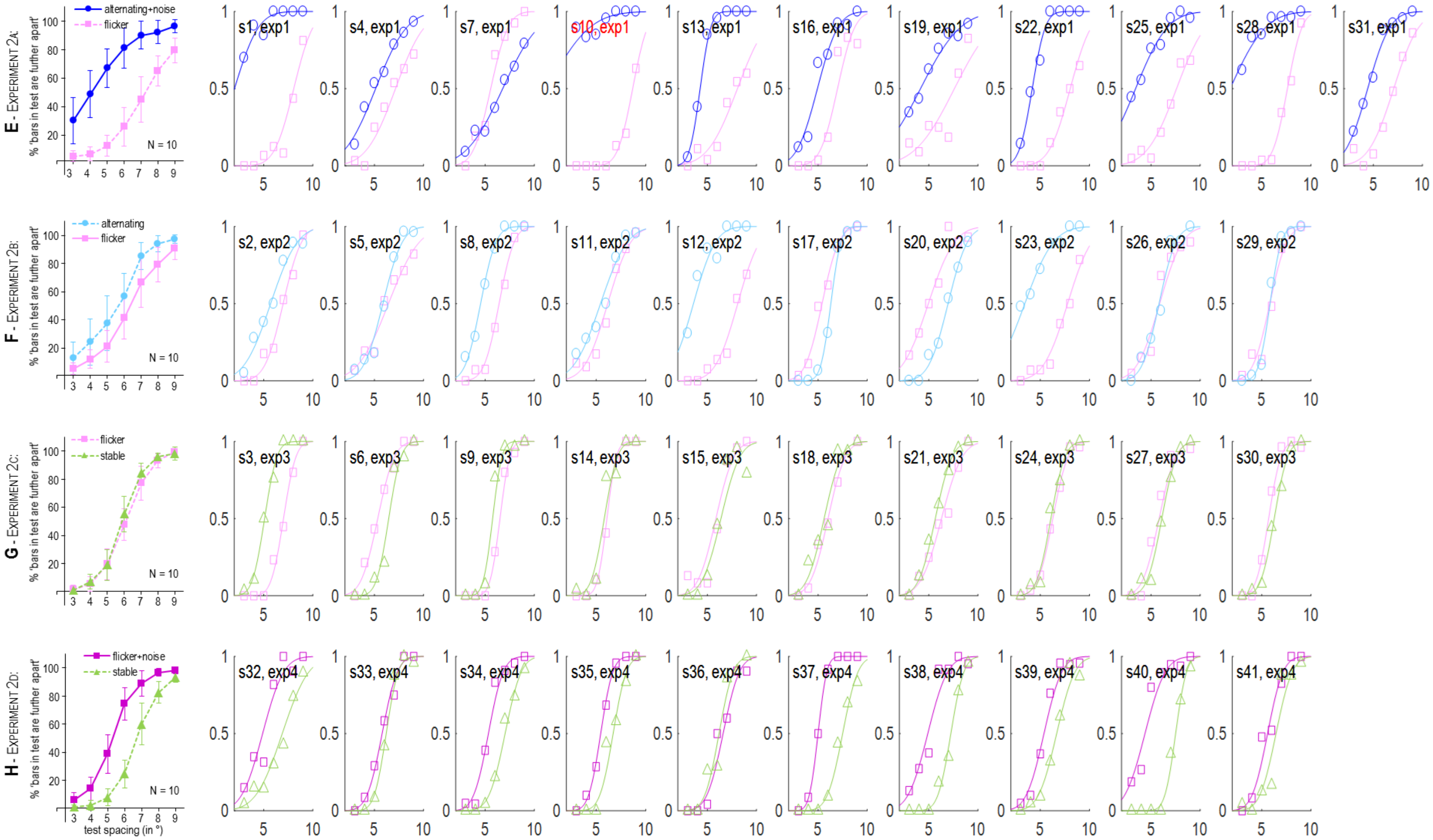


**Supplementary Figure S2.** Proportions of 'bars in test further apart' responses individually for all participants from Experimental Series 2, along with the psychometric functions fit to the data. Leftmost column: averaged data as reported in the manuscript. Note that the participant excluded from analysis in Exp2a (s10; marked in red font) shows a pattern similar to the averaged data.

## EXPERIMENTAL SERIES 3: STABLE BARS AND/OR NOISE

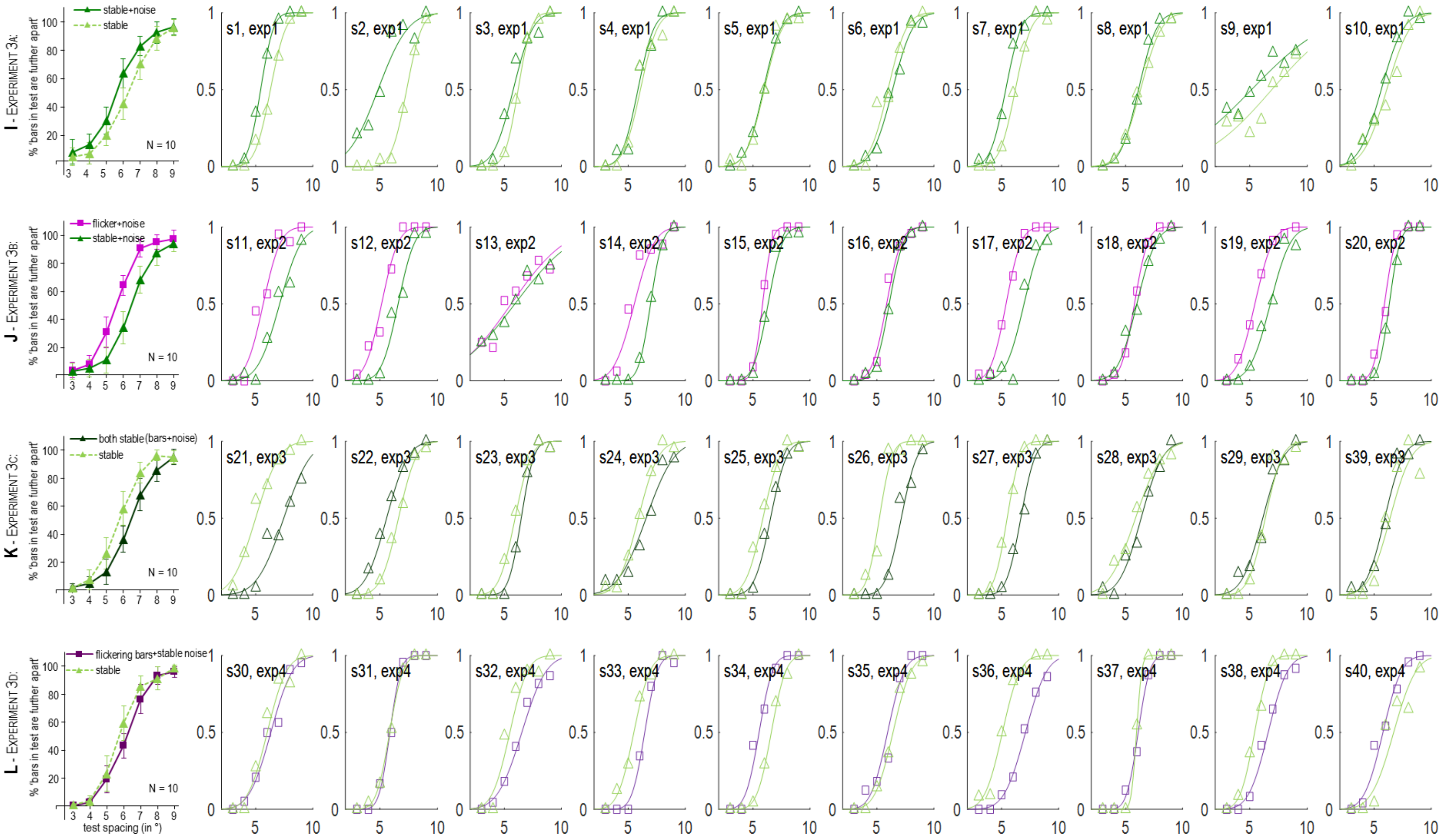


Supplementary Figure S3. Proportions of 'bars in test further apart' responses individually for all participants from Experimental Series 3, along with the psychometric functions fit to the data. Leftmost column: averaged data as reported in the manuscript. No datasets were excluded from analysis.
