# Supplementary material for: Repeatedly Flashed Luminance Noise Can Make Objects Look Further Apart
Source: Iperception. 2019 Jun 20;10(3):2041669519855090. doi: 10.1177/2041669519855090 (PMC6591532; doi:10.1177/2041669519855090)
Supplement: Supplementary material [file Supplemental_Material2.pdf]

Supplementary Series 101: Alternating Bars with Full-Screen Luminance Noise

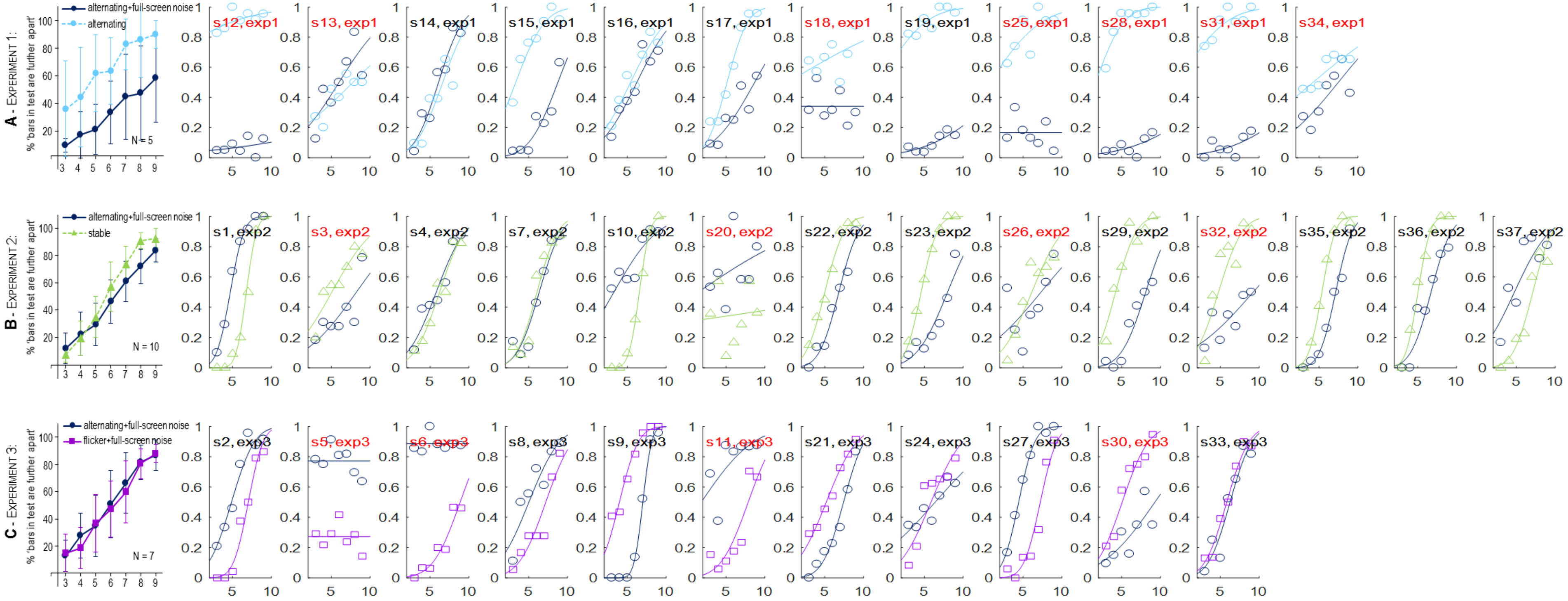

Supplementary Figure S4. Proportions of 'bars in test further apart' responses individually for all participants from Supplementary Series 101, along with the psychometric functions fit to the data. Participants whose data was excluded from analysis (due to R^2 of at least one of the fits < 0.7) are marked in red font. Leftmost column: averaged data for the remaining participants. Exp1d reported in the manuscript was actually part of this series (Exp 2, middle row). Alternating bars embedded in full-screen luminance noise were compared to stimulus streams in which bars were not embedded in the noise pattern (by leaving a small 'window'; Exp1: alternating, see demo here; Exp2: stable, see demo here) or flickering stimuli likewise embedded in the full-screen noise pattern (Exp3: see demo here). A relatively large number of participants had to be excluded, suggesting that the used procedure and subsequent analysis were inappropriate for these conditions. In Exp1 most of the excluded participant showed a similar pattern, reporting the alternating bars without luminance noise as further apart, across all spacings. This would be consistent with our previous studies where we found full-screen noise patterns to shrink the perceived distance between stimuli ('masked-induced compression of space'; e.g., Born et al., 2016). However, to confirm this, different methods would need to be applied: with the current methodology, on the one hand, excluding half the participants from analysis is problematic, especially when they all show a very similar pattern; on the other hand, PSEs are not interpretable with such large differences. Exp3 did not produce that many extremes, but rather inconsistent data across participants. Importantly, whether this comparison may eventually produce a consistent bias or not is not particularly crucial for the current study. Exp1 and Exp3 are therefore not reported in the manuscript and I made no further effort to obtain ten acceptable data sets for analysis. To acknowledge, however, that the reported biases seem to be specific to the small 'boxed' noise patterns used in the other conditions, Exp2 is reported in the manuscript as Exp1d and data was collected until ten acceptable data sets were obtained.

Supplementary Series 102: Flickering Bars with Full-Screen Luminance Noise

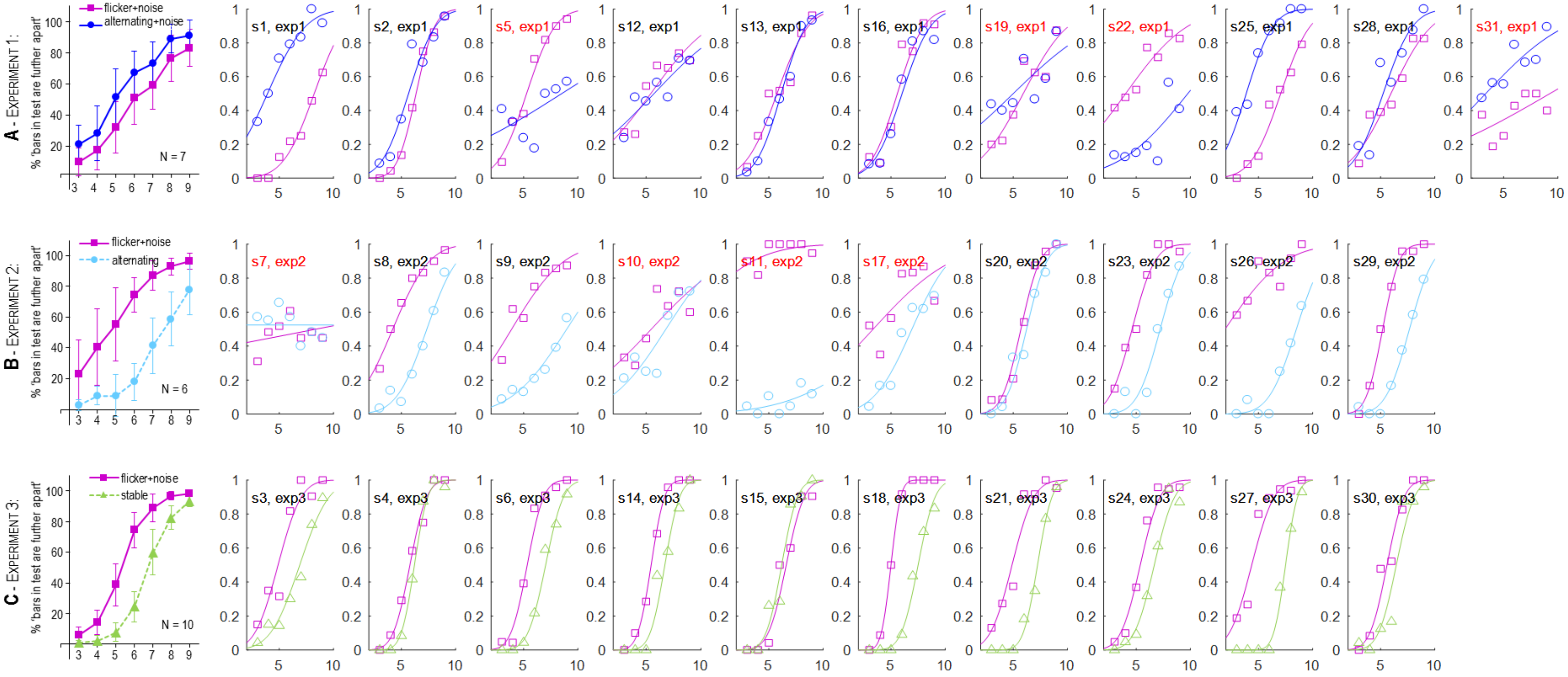

Supplementary Figure S5. Proportions of 'bars in test further apart' responses individually for all participants from Supplementary Series 102, along with the psychometric functions fit to the data. Participants whose data was excluded from analysis (due to R^2 of at least one of the fits < 0.7) are marked in red font. Leftmost column: averaged data for the remaining participants. Note that Experiment 2d reported in the manuscript was actually part of this series (Experiment 3, last row). In Exp1, flickering bars with luminance noise were compared to alternating bars with luminance noise (see demo here). Results hint towards small overestimations on the alternating side. This is reminiscent of the effect found in Exp3b where flickering bars with luminance noise produced overestimations compared to the stable condition with luminance noise: in both experiments, when the noise pattern is presented on both sides, the less continuous stimulus is chosen more frequently. However, the data is rather variable across participants and statistics are likely to remain inconclusive even with ten participants. Importantly, whether this comparison produces a consistent bias or not is not crucial for this study. Therefore this comparison is not reported in the manuscript. Results of Exp2, on the other hand, are perfectly in line with the main conclusion put forth in the manuscript: when flickering bars with luminance noise are compared to alternating bars without noise (see demo here), participants are biased to report the bars with luminance noise as appearing further apart (all but one participant showed such a pattern). The comparison was not pursued until ten acceptable data sets were obtained and omitted from the manuscript simply for brevity.
