# Supplementary figures and images for: Repeatedly Flashed Luminance Noise Can Make Objects Look Further Apart
Source: Iperception. 2019 Jun 20;10(3):2041669519855090. doi: 10.1177/2041669519855090 (PMC6591532; doi:10.1177/2041669519855090)

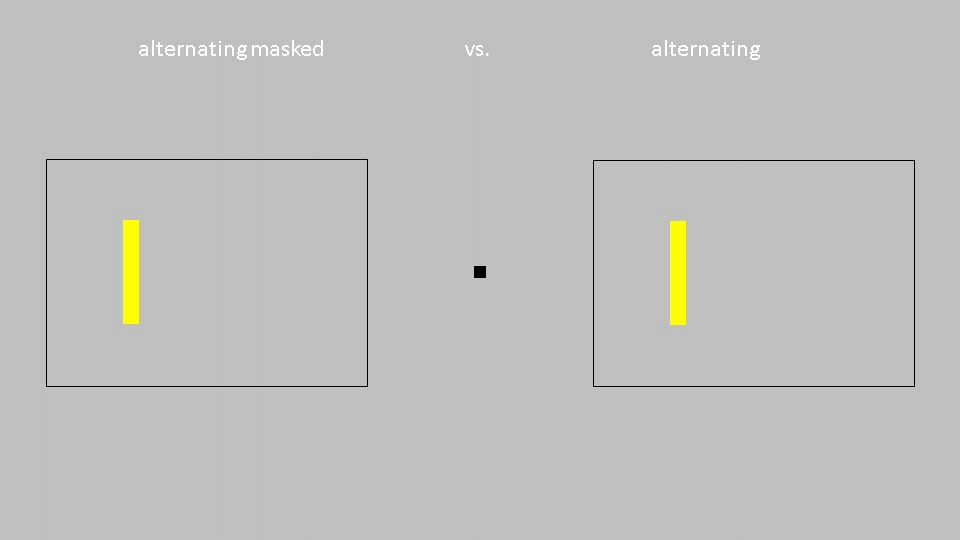

Supplement: Supplementary material [file Supplemental_Movie1a.gif]

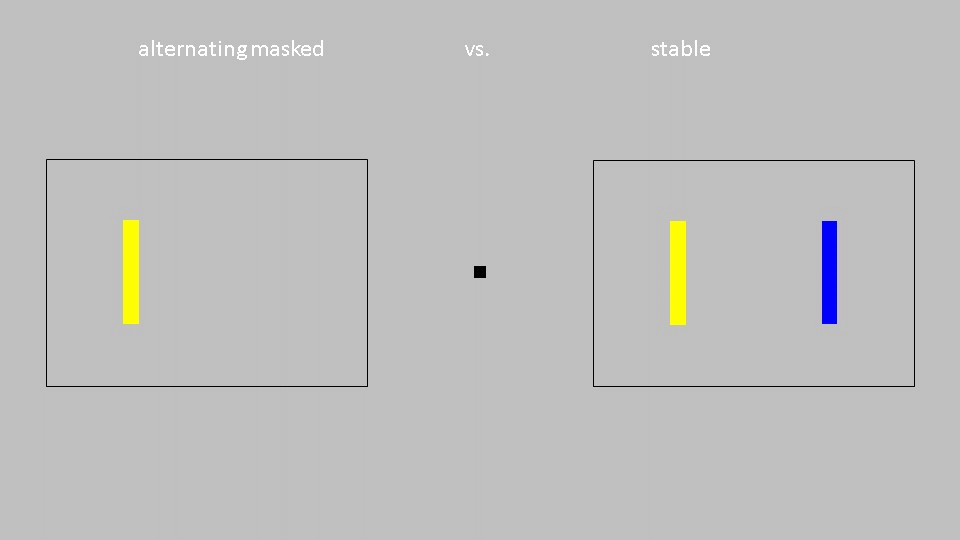

Supplement: Supplementary material [file Supplemental_Movie1b.gif]

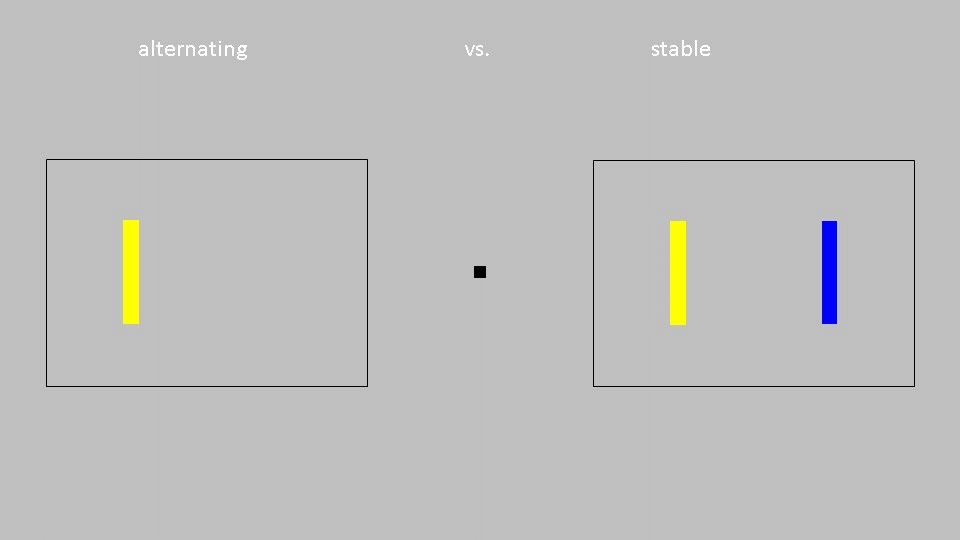

Supplement: Supplementary material [file Supplemental_Movie1c.gif]

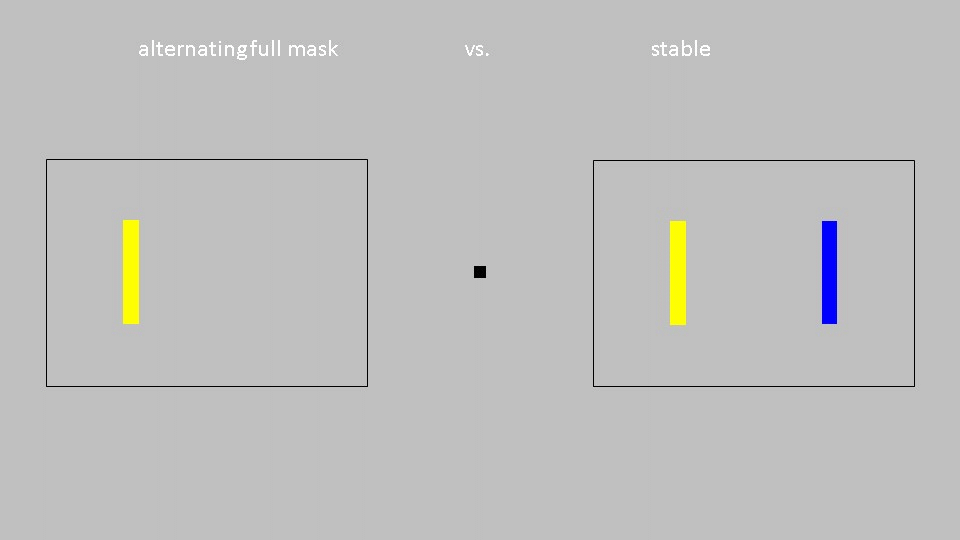

Supplement: Supplementary material [file Supplemental_Movie1d.gif]

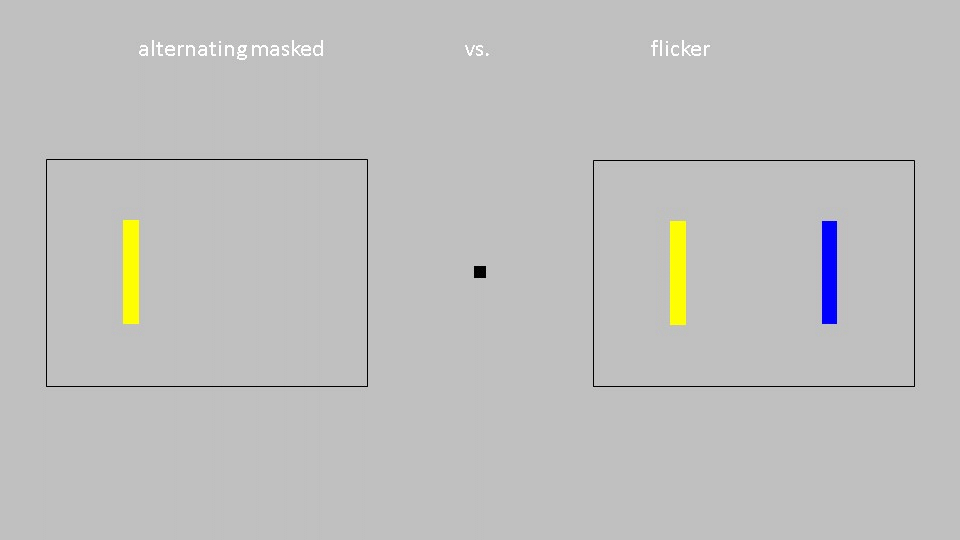

Supplement: Supplementary material [file Supplemental_Movie2a.gif]

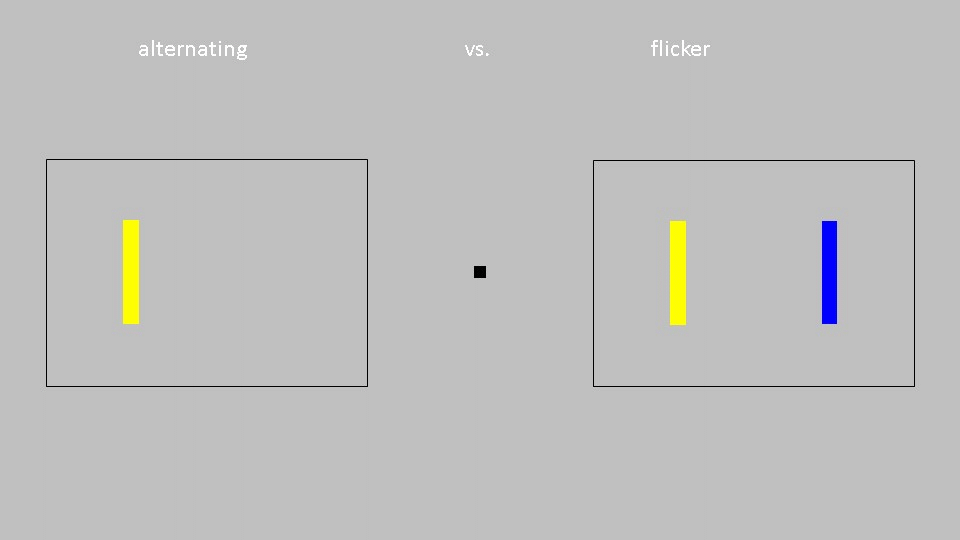

Supplement: Supplementary material [file Supplemental_Movie2b.gif]

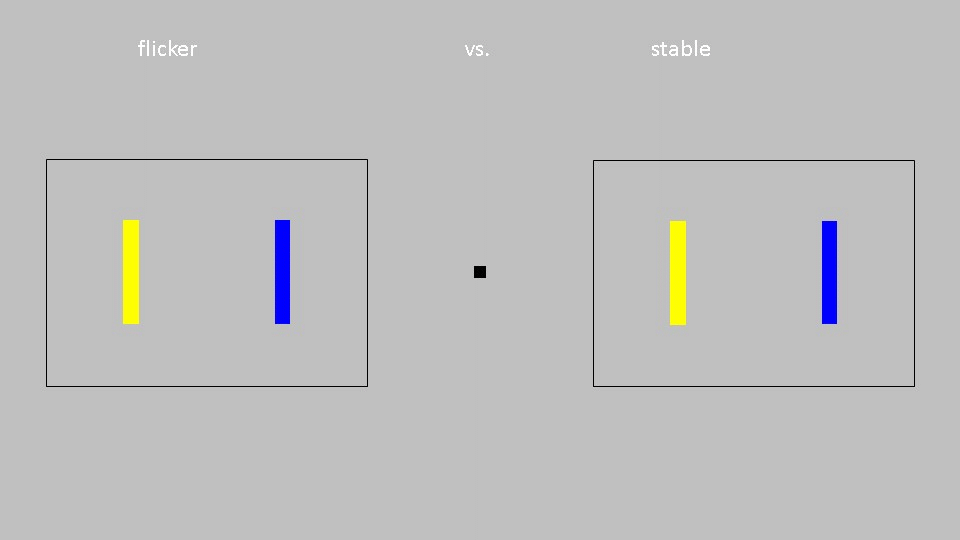

Supplement: Supplementary material [file Supplemental_Movie2c.gif]

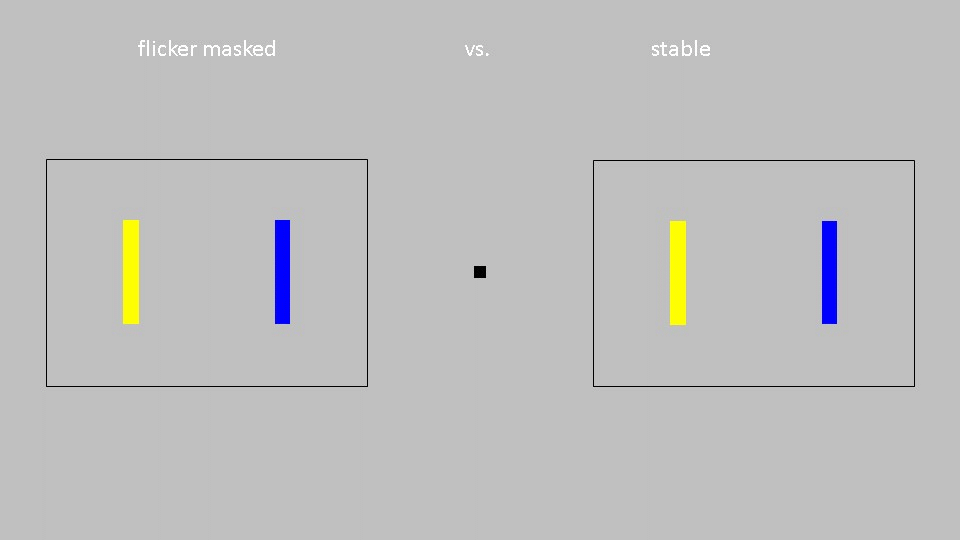

Supplement: Supplementary material [file Supplemental_Movie2d.gif]

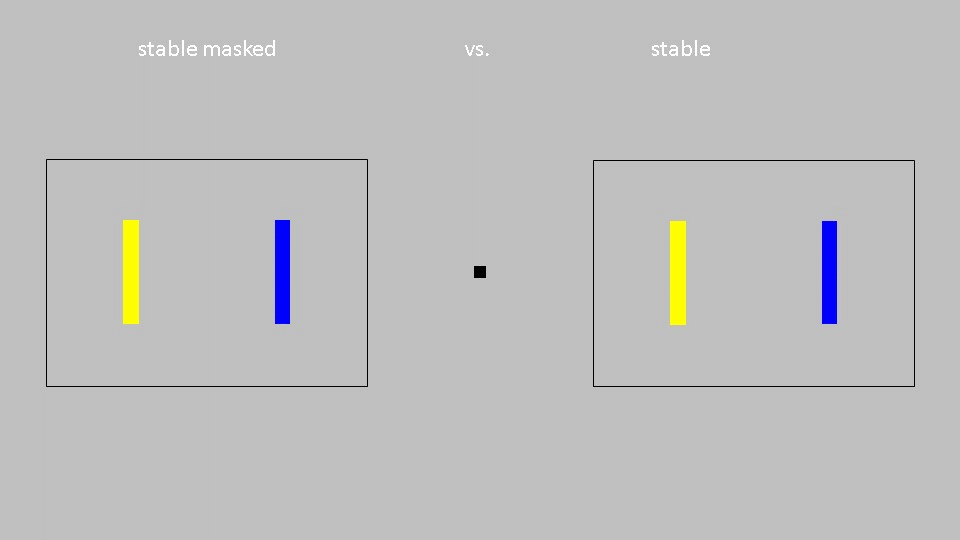

Supplement: Supplementary material [file Supplemental_Movie3a.gif]

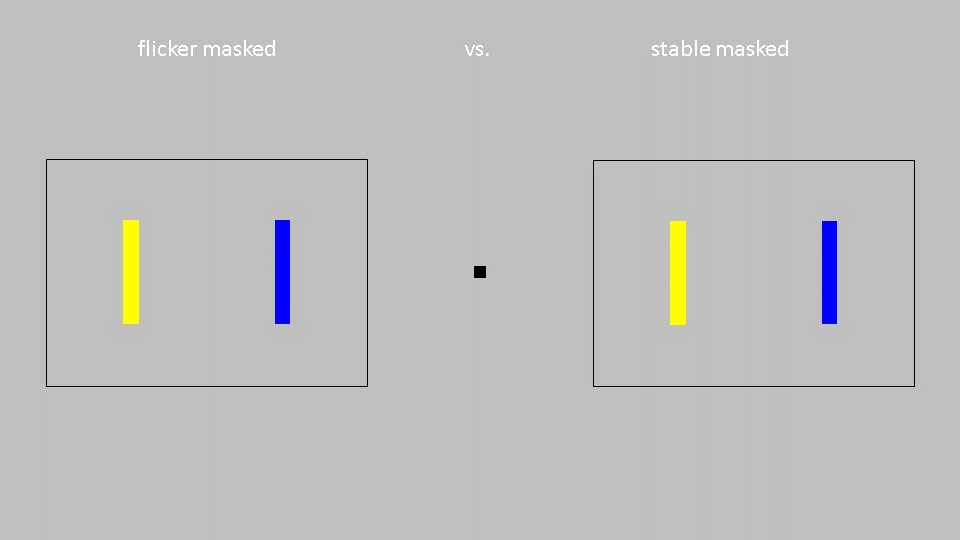

Supplement: Supplementary material [file Supplemental_Movie3b.gif]

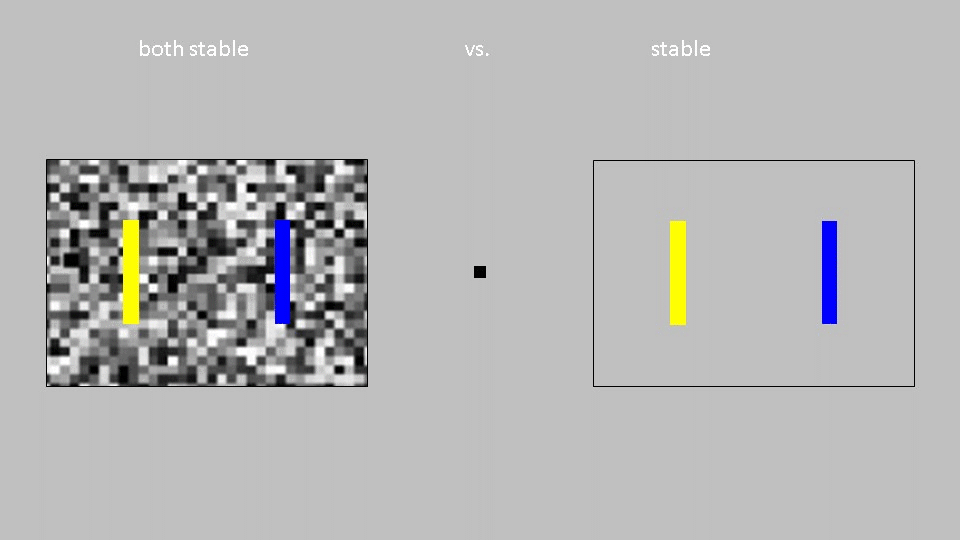

Supplement: Supplementary material [file Supplemental_Movie3c.gif]

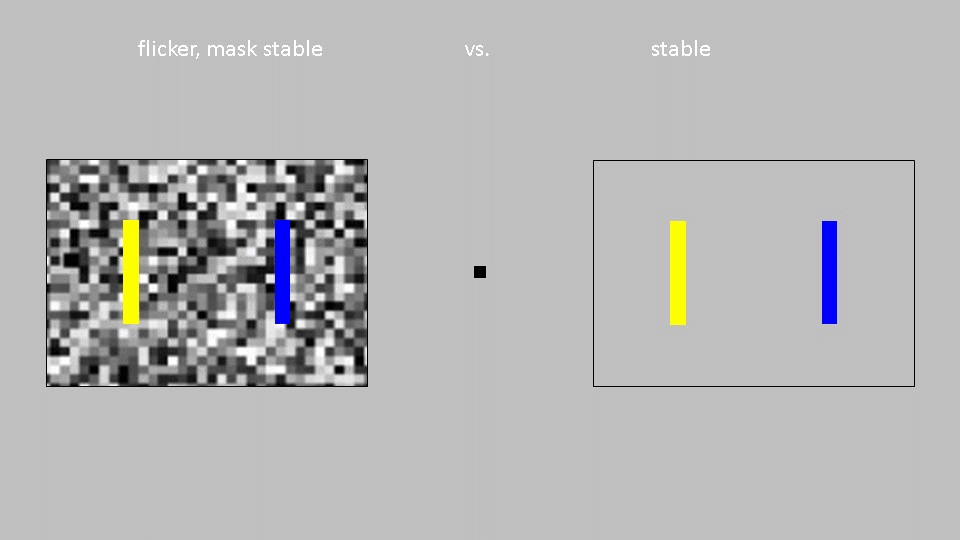

Supplement: Supplementary material [file Supplemental_Movie3d.gif]

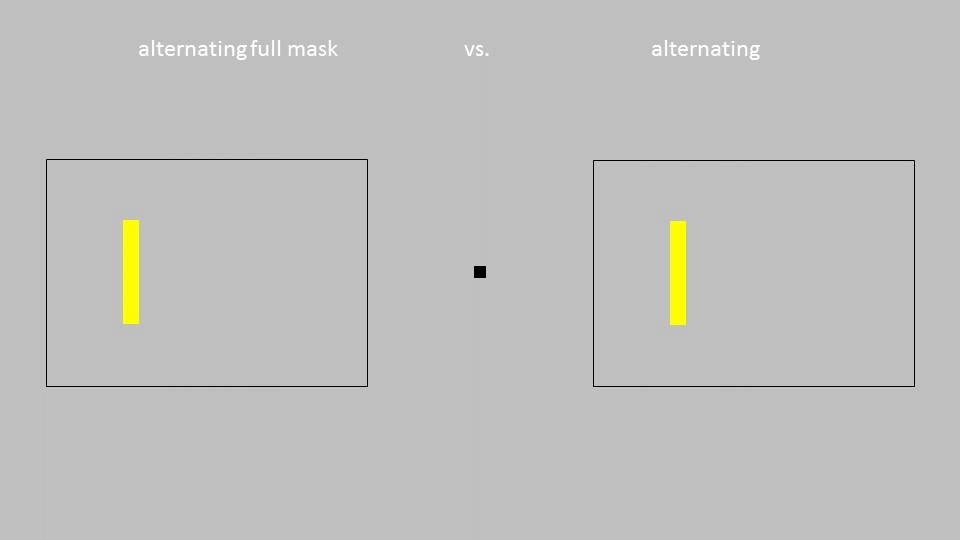

Supplement: Supplementary material [file Supplemental_Movie101_1.gif]

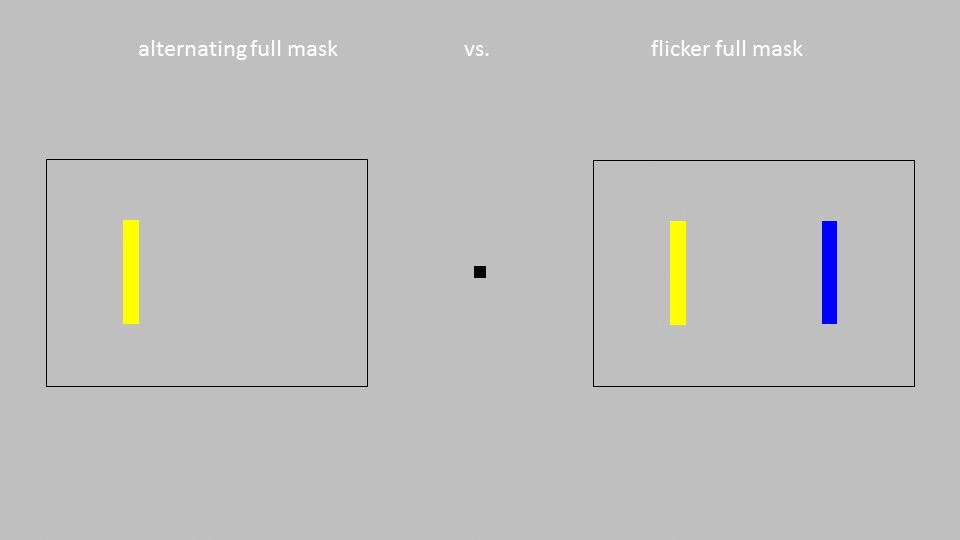

Supplement: Supplementary material [file Supplemental_Movie101_3.gif]

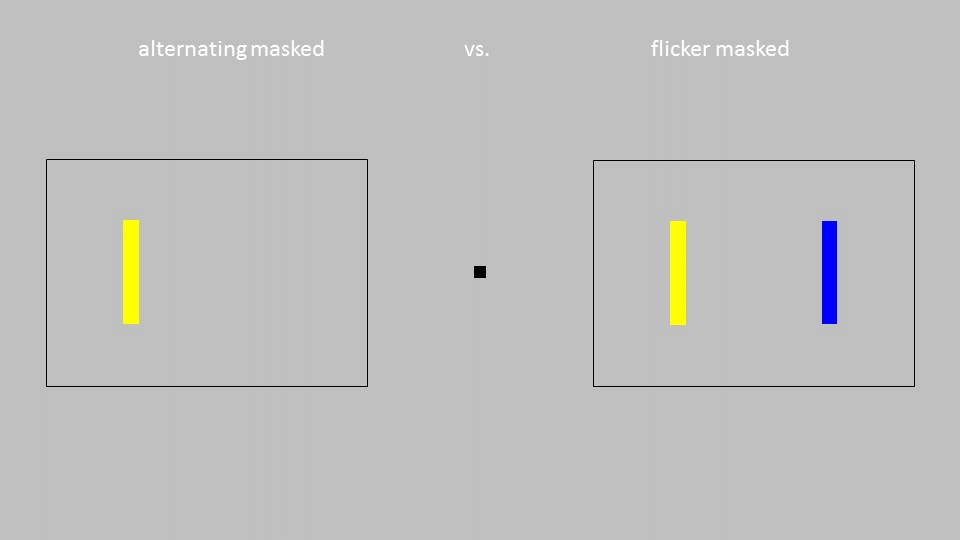

Supplement: Supplementary material [file Supplemental_Movie102_1.gif]

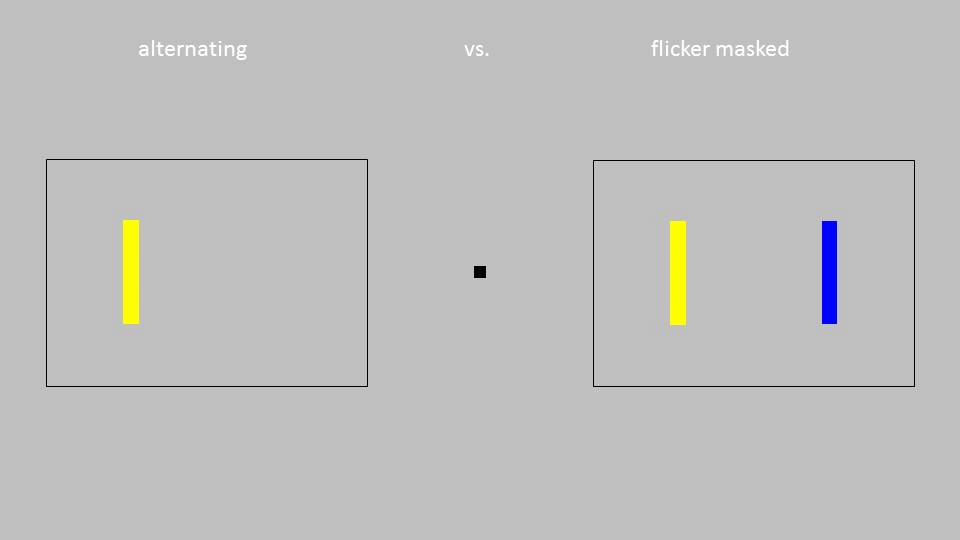

Supplement: Supplementary material [file Supplemental_Movie102_2.gif]
